# ^68^Ga-DOTATATE PET/CT versus ^111^In-octreotide scintigraphy in patients with neuroendocrine tumors: a prospective study

**DOI:** 10.1590/0100-3984.2021.0038

**Published:** 2022

**Authors:** Marcelo Cavicchioli, Almir Galvão Vieira Bitencourt, Eduardo Nóbrega Pereira Lima

**Affiliations:** A.C. Camargo Cancer Center – Imaging Department, São Paulo, SP, Brazil.

**Keywords:** Carcinoma, neuroendocrine/diagnostic imaging, Positron-emission tomography/methods, Tomography, X-ray computed/ methods, Radionuclide imaging/methods, Carcinoma neuroendócrino/diagnóstico por imagem, Tomografia por emissão de pósitrons/métodos, Tomografia computadorizada/métodos, Cintilografia/métodos

## Abstract

**Objective:**

To compare ^68^Ga-DOTA-DPhe1,Tyr3-octreotate (^68^Ga-DOTATATE) positron-emission tomography/computed tomography (PET/CT) findings with those of conventional ^111^In-octreotide scintigraphy in patients with neuroendocrine tumors (NETs).

**Materials and Methods:**

This was a single-center prospective study including 41 patients (25 males; mean age, 55.4 years) with biopsy-proven NETs who underwent whole-body ^111^In-octreotide scintigraphy and whole-body ^68^Ga-DOTATATE PET/CT. The patients had been referred for tumor staging (34.1%), tumor restaging (61.0%), or response evaluation (4.9%). Images were compared in a patient-by-patient analysis to identify additional lesions, and we attempted to determine the impact that discordant findings had on treatment planning.

**Results:**

Compared with ^111^In-octreotide scintigraphy, ^68^Ga-DOTATATE PET/CT revealed more lesions, the additional lesions typically being in the liver or bowel. Changes in management owing to the additional information provided by ^68^Ga-DOTATATE PET/CT occurred in five patients (12.2%), including intermodal changes in three (7.3%) and intramodal changes in two (4.9%). In addition, ^68^Ga-DOTATATE PET/CT yielded incidental findings unrelated to the primary NET in three patients (7.3%): Hürthle cell carcinoma of the thyroid, bowel non-Hodgkin lymphoma, and a suspicious breast lesion.

**Conclusion:**

We conclude that ^68^Ga-DOTATATE PET/CT is superior to conventional ^111^In-octreotide scintigraphy for the management of NETs because of its ability to determine the extent of the disease more accurately, which, in some cases, translates to changes in the treatment plan.

## Abstract (pt)

**Objetivo:**

Comparar os achados da PET/CT com ^68^Ga-DOTATATE em relação aos da cintilografia com ^111^In-octreotide em pacientes com tumores neuroendócrinos (TNEs).

**Materiais e Métodos:**

Estudo prospectivo unicêntrico incluindo 41 pacientes (25 homens; média de idade: 55,4 anos) com TNEs comprovados por biópsia submetidos a cintilografia de corpo inteiro com ^111^In-octreotide e PET/CT com ^68^Ga-DOTATATE. Os pacientes incluídos foram encaminhados para estadiamento do tumor (34,1%), reestadiamento (61,0%) ou avaliação da resposta (4,9%). As imagens foram comparadas para identificar lesões adicionais e o impacto dos achados discordantes no planejamento terapêutico.

**Resultados:**

Na comparação com a cintilografia com ^111^In-octreotide, a PET/CT com ^68^Ga-DOTATATE revelou mais lesões, mais frequentemente localizadas no fígado e intestino. Mudanças no tratamento devidas às informações adicionais reveladas pela PET/ CT ocorreram em 5/41 pacientes (12,2%), incluindo mudanças intermodalidade em três casos (7,3%) e intramodalidade em dois casos (4,9%). A PET/CT também identificou achados incidentais não relacionados ao TNE em 3/41 pacientes (7,3%), incluindo um carcinoma de células de Hürthle da tireoide, um linfoma não Hodgkin de intestino e uma lesão mamária suspeita.

**Conclusão:**

A PET/CT com ^68^Ga-DOTATATE é superior à cintilografia convencional com ^111^In-octreotide para o manejo de pacientes com TNEs, em virtude da sua capacidade de detectar a extensão da doença com mais precisão, o que se traduz, em alguns casos, em alterações terapêuticas.

## INTRODUCTION

Although neuroendocrine tumors (NETs) constitute a rare type of tumor, the rate of their detection has been increasing substantially because of the ever greater accuracy of diagnostic modalities. They originate mainly in the gastrointestinal tract or lung tissues and are characterized by somatostatin receptor (SSTR) overexpression. The treatment of a NET should be individualized and often requires a multimodal approach, which may include some combination of surgery, molecularly targeted therapy, chemotherapy, somatostatin analogue radiopharmaceutical therapy, ablation, and embolization. The localization of primary tumors and detection of metastatic lesions are essential to proper therapeutic planning(^1^,^2^,^3^).

It is often difficult to locate NETs by conventional imaging because of their small size, multiplicity, and presence in the gastrointestinal tract. In recent decades, scintigraphy with ^111^In-radiolabeled somatostatin analogues has proven to be an effective diagnostic imaging method for the detection of well-differentiated NETs.

The introduction of hybrid systems, such as singlephoton emission computed tomography/computed tomography (SPECT/CT) has improved the localization of lesions identified by SSTR scintigraphy (SRS) with a somatostatin analogue. Although SPECT/CT shows high efficacy for whole-body imaging, it has limitations in organs with higher physiological uptake, such as the liver, and in the detection of small lesions due to the low spatial resolution of the method(^4^,^5^,^6^,^7^,^8^).

More recently, positron emission tomography/CT (PET/CT) employing ^68^Ga- DOTA-DPhe^1^,Tyr^3^-octreotate (^68^Ga-DOTATATE)-radiolabeled SSTRs has been used to overcome those limitations. This method provides higher resolution and has a better pharmacokinetic profile than does SRS. The proportion of cases in which PET/CT findings lead to changes in treatment in patients with a NET is quite variable, ranging from 16% to 71%(^9^,^10^,^11^,^12^,^13^,^14^,^15^,^16^,^17^,^18^,^19^,^20^,^21^,^22^).

The objective of the present study was to compare ^68^Ga-DOTATATE PET/CT with conventional ^111^In-octreotide SRS for lesion detection in patients diagnosed with a NET, as well as to evaluate its impact on treatment.

## MATERIALS AND METHODS

### Patients

This was a cross-sectional, prospective, single-center study involving a sample of 41 consecutive patients (25 males and 16 females), with a mean age of 55.4 years (range, 24–86 years). All of the patients had a biopsyproven NET and underwent both ^68^Ga-DOTATATE PET/ CT and conventional ^111^In-octreotide SRS between April 2014 and October 2016. The study protocol was approved by the local institutional review board, and all participating patients gave written informed consent.

All of the patients had been referred to our department for tumor staging (34.1%), tumor restaging (61.0%), or response evaluation (4.9%). Most of the NETs were well-differentiated, the classification being grade 1 in 29 (70.7%) and grade 2 in eight (19.5%), whereas poorly differentiated (grade 3) NETs were seen in four cases (9.8%). The origins of the primary tumors were as follows: gastro-enteropancreatic, in 31 cases (75.6%); pancreatic, in 15 (36.6%); unknown, in seven (17.1%); pulmonary, in two (4.9%); and biliary, in one (2.4%).

Of the 41 patients evaluated, 27 (65.9%) had previously been treated, including 10 (24.4%) who had been submitted to at least three different treatment modalities. Prior treatments included surgical resection of the primary tumor (in 34.4%), somatostatin analogue therapy (in 29.7%), chemoembolization/embolization (in 12.5%), ^177^Lu-octreotate treatment (in 9.4%), molecularly targeted therapy (in 7.8%), and chemotherapy (in 6.2%).

### Image acquisition

All of the patients underwent conventional ^111^In-octreotide SRS followed within 15 days by ^68^Ga-DOTATATE PET/CT. Administration of short-acting octreotide or a long-acting somatostatin analogue was discontinued 10 and 30 days prior to scanning, respectively.

Conventional ^111^In-octreotide SRS protocols included planar scans and SPECT/CT, when possible. Conventional ^111^In-octreotide SRS was performed after intravenous administration of 185 MBq of ^111^In-pentetreotide (OCTIPEN; Instituto de Pesquisas Energéticas e Nucleares, São Paulo, Brazil). Images were acquired with a dual-head, large field-of-view gamma camera (Optima NM/CT 640; GE Healthcare, Milwaukee, WI, USA) equipped with a medium-energy collimator. Planar whole-body scans were acquired at 3–4 h and 24 h after radiopharmaceutical administration. Eight patients also underwent an abdominal SPECT/CT examination at 4 h after radiopharmaceutical administration.

Images were reconstructed iteratively based on the ordered subset expectation maximization algorithm. Transmission data obtained during CT were used for anatomical localization of scintigraphic findings. For the assessment of radiotracer uptake, ^111^In-octreotide intensities of tumor foci were compared with physiological liver uptake intensities observed on a planar whole-body scan. The intensities seen on attenuation-corrected SPECT/CT were also taken into account.

All PET/CT scans were acquired in a fully 3-dimensional scanner (Gemini TF; Philips Medical Systems, Cleveland, OH, USA). The ^68^Ga-DOTATATE was administered intravenously, scanning beginning 50–60 min after injection of 185 MBq of the radiotracer. The PET/CT protocol consisted of an unenhanced CT scan followed by a PET scan. The PET images were acquired from the top of the skull to the mid-thigh, with a 3-min bed positioning time and the patient lying supine with the arms down and extended. The PET images were corrected for tissue attenuation based on CT data, after which they were reconstructed by iterative reconstruction. The same images were then evaluated qualitatively and semi-quantitatively based on a standard uptake value, defined as the quotient of maximum activity concentration divided by the ratio between the injected dose (numerator) and the body weight of the patient.

### Image analysis

The ^68^Ga-DOTATATE PET/CT and ^111^In-octreotide SRS images were reviewed and interpreted by two nuclear medicine physicians, working independently, each blinded to the other set of images and to other imaging modalities. Any area with an intensity greater than the background not attributable to physiological activity was considered suspicious for tumor tissue. The ^68^Ga-DOTATATE PET/CT and conventional ^111^In-octreotide SRS findings in each region were classified as concordant if the same foci were seen in that region on both modalities or discordant if foci were seen on only one modality. In cases of discrepancy, the images were reviewed by a third nuclear medicine physician. After individual patient analysis of the imaging findings, their effects on the treatment were assessed. Changes in management were further classified as intermodal (e.g., from surgery to systemic therapy) or intramodal (e.g., a change in the extent of surgery or in the chemotherapy regimen). The impact of imaging-study monitoring was classified as low, moderate, or high based on the complexity of the change in treatment proposed after the examination.

Data analysis

All analyses were performed with the IBM SPSS Statistics software package, version 20.0 (IBM Corp., Armonk, NY, USA). Categorical variables were reported as absolute and relative frequencies. Continuous variables were reported as means and standard deviations. Pearson chi-square tests with Yates correction or Fisher’s exact tests were used in order to analyze case management changes in relation to categorical clinical variables. Values of *p* ≤ 0.05 were considered statistically significant.

## RESULTS

**Figure 1:**
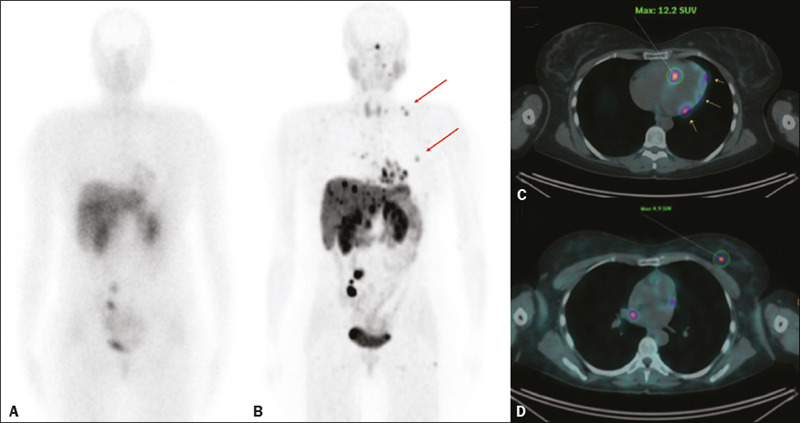
^111^In-octreotide SRS and ^68^Ga-DOTATATE PET/CT images obtained in the case of a 45 year-old woman being evaluated for NET metastases. **A:** ^111^In-octreotide SRS (anterior plane, 24 h after radiotracer administration) showing lesions in the mediastinum, myocardium, liver, and bowel. **B:** ^68^Ga-DOTATATE PET/CT (maximum-intensity-projection image) showing better delineation of the lesions shown in **A,** as well as additional lesions in the left breast and lower neck (arrows). **C,D:** Axial PET/CT images showing abnormal uptake in the myocardium (**C**) and left breast (**D**).

The results of the ^111^In-octreotide SRS and ^68^Ga-DOTATATE PET/CT were concordant in 36 (87.8%) of the 41 patients evaluated, both being positive in 33 (80.5%) and both being negative in three (7.3%). The results were discordant in the five remaining patients (12.2%), all of whom had a positive PET/CT result and a negative SRS result, including three patients with liver metastases and two patients with pancreatic tumors. Among the 33 patients in which the results were positive in both modalities, ^68^Ga-DOTATATE PET/CT revealed additional tumor sites in 11: in the bowel (n = 3); in lymph nodes (n = 2); in the liver (n = 1); in bone (n = 1); in the pancreas (n = 1); in the orbit (n = 1); in the breast (n = 1); and in the thyroid (n = 1). Images from an example case in which ^68^Ga-DOTATATE PET/CT revealed additional tumors are shown in Figure 1. Overall, the ^68^Ga-DOTATATE PET/CT imaging revealed more tumor sites than did ^111^In-octreotide SRS. The locations of the lesions detected only by PET/CT were, in decreasing order, the liver, bowel, lymph nodes, bone, and pancreas (Table 1).

**Table:** Positive results obtained with ^68^Ga-DOTATATE PET/CT and ^111^In-octreotide SRS, together with additional lesions revealed by PET/CT, by tumor site.

Tumor site	Patients with positive PET/CT results	Patients with positive SRS results	Patients with additional lesions revealed by PET/CT
	n	n	n
Liver	27	23	23
Bowel	13	9	11
Lymph node(s)	21	10	9
Bone	9	7	7
Pancreas	9	6	2

Changes in case management based on the additional information obtained by ^68^Ga-DOTATATE PET/CT occurred in five (12.2%) of the 41 patients (Table 2): inter-modal changes (additional radiotherapy for an intraconal NET, surgery for a second primary pancreatic NET, or the introduction of bisphosphonates) in three; and intramodal changes (more extensive hepatectomy and the addition of hepatic nodulectomy) in two. Changes in management were not significantly related to age, gender, indication for the examination, tumor grade, primary tumor site, or prior treatment.

**Table:** Summary of treatment changes enacted based on additional positive ^68^Ga-DOTATATE PET/CT findings (n = 5).

Additional finding	N	Treatment change	Change	
			Class	Impact
Liver metastases	2	More extensive hepatectomy; addition of hepatic nodulectomy	Intramodal	Moderate
Suspected primary tumor	1	Resection of a second primary pancreatic tumor	Intermodal	High
Bone metastasis	1	Introduction of bisphosphonates	Intermodal	Low
Orbital metastasis	1	Additional radiotherapy for an intraconal NET	Intermodal	Low

The ^68^Ga-DOTATATE PET/CT yielded incidental findings unrelated to the primary NET in three (7.3%) of the 41 patients. One patient was found to have a second primary thyroid tumor, which was confirmed postoperatively to be Hürthle cell carcinoma; one had bowel non-Hodgkin lymphoma, which was also confirmed postoperatively; and one had a suspected second primary breast tumor, which was confirmed by percutaneous biopsy to be a metastatic NET.

## DISCUSSION

Our data demonstrate the superiority of ^68^Ga-DOTATATE PET/CT over ^111^In-octreotide SRS for the detection of NETs. Although most of the findings were concordant between the two methods, ^68^Ga-DOTATATE PET/CT yielded additional findings that served to indicate changes in the treatment plan in 12.2% of the cases evaluated. Even when both modalities detected the same tumor sites, ^68^Ga-DOTATATE PET/CT demonstrated the location of those sites with better precision and greater radiotracer uptake.

The present study expands upon previous reports demonstrating the superiority of ^68^Ga-DOTATATE PET/ CT over SRS (including ^111^In-octreotide SRS) and other conventional anatomical imaging modalities (CT and magnetic resonance imaging) for the evaluation of NETs. However, the detection of a greater number of lesions is not necessarily followed by a change in the disease staging or treatment plan^(23)^. Findings that may lead to changes in the treatment plan include the detection of previously unsuspected local recurrence or metastasis, the identification of occult primary tumors, and the confirmation of SSTR expression by tumors(^11^,^23^,^24^). High-impact changes to the treatment plan include expanding the extent of the surgery planned, the addition of putatively curative resection of a NET of unknown origin, and the addition of systemic therapy due to detection of multiple metastases(^25^,^26^).

Among the five cases in which ^68^Ga-DOTATATE PET/ CT led to changes in the NET treatment plan in our sample, the changes were intermodal in three and intramodal in two. The rate at which a discordant result led to a change in the treatment plan was lower in the present study than in several previous studies, probably because of the high prevalence of patients with metastatic disease in our sample. The reported frequency of therapeutic changes varies widely in the literature, ranging from 4% to 83%, which reflects substantial heterogeneity across studies in terms of patient selection, methods, and the definition of changes to the treatment plan(^10^,^21^,^26^,^27^,^28^). Considering only studies in which PET/CT was performed after ^111^In-octreotide scanning, one meta-analysis showed that changes in management were observed, on average, in 39% of patients (range, 16–71%), most (77%) being intermodal changes(^12^,^19^,^20^,^21^,^25^).

We observed another advantage of ^68^Ga-DOTATATE PET-CT with respect to incidental findings unrelated to the primary NET, which has not commonly been described in the literature. In our sample, such findings included a thyroid nodule confirmed to be a second primary tumor (Hürthle cell carcinoma), a mesenteric mass confirmed to be a sign of synchronous lymphoproliferative disease (mantle lymphoma), and a small breast mass confirmed to be a NET metastasis after a primary breast tumor was ruled out.

Our study has some limitations. First, we investigated a heterogeneous group of patients in different stages of the evolution of disease. However, this heterogeneity is representative of real-world clinical populations of patients undergoing ^68^Ga-DOTATATE PET/CT. Second, we did not perform a lesion-by-lesion comparative analyses because not all patients were submitted to SPECT/CT studies during ^111^In-octreotide SRS. That is a significant limitation because SPECT/CT images are more sensitive than are planar and SPECT images alone, as well as providing better anatomical correlation of the findings^(29)^. In addition, we evaluated only changes to the treatment plan that were prompted by discordant findings. The clinical oncology team accessing the data obtained from these diagnostic imaging studies made treatment decisions based on those findings together with other clinical observations, including previously collected morphological information, as has been the case in other studies(^17^,^25^,^26^).

Although ^68^Ga-DOTATATE PET/CT is not as widely available as is ^111^In-octreotide SRS, the costs of the two methods are comparable. Therefore, we believe that, over time, ^68^Ga-DOTATATE PET/CT is likely to replace ^111^In-octreotide SRS for the monitoring of patients with NETs. Together with ^18^F-fluorodeoxyglucose PET/CT, ^68^Ga-DOTATATE PET/CT can be used in order to assess heterogeneity of a NET noninvasively and to improve their individualized management(^30^,^31^).

In conclusion, ^68^Ga-DOTATATE PET/CT is clinically superior to ^111^In-octreotide SRS for the management of NETs because of its ability to detect the extent of disease more accurately, which, in some cases, translates to changes in the treatment plan. In addition, ^68^Ga-DOTATATE PET/ CT may improve patient care by yielding additional incidental findings. Our findings highlight the importance of incorporating this method into the routine evaluation of patients with NETs.

## References

[1] Barakat MT, Meeran K, Bloom SR (2004). Neuroendocrine tumours. Endocr Relat Cancer.

[2] Modlin IM, Oberg K, Chung DC (2008). Gastroenteropancreatic neuroendocrine tumours. Lancet Oncol.

[3] Basuroy R, Srirajaskanthan R, Ramage JK (2016). Neuroendocrine tumors. Gastroenterol Clin North Am.

[4] Gotthardt M, Dijkgraaf I, Boerman OC (2006). Nuclear medicine imaging and therapy of neuroendocrine
tumours. Cancer Imaging.

[5] Kaltsas G, Rockall A, Papadogias D (2004). Recent advances in radiological and radionuclide imaging and
therapy of neuroendocrine tumours. Eur J Endocrinol.

[6] Koopmans KP, Neels ON, Kema IP (2009). Molecular imaging in neuroendocrine tumors: molecular uptake
mechanisms and clinical results. Crit Rev Oncol Hematol.

[7] Kwekkeboom DJ, Krenning EP, Lebtahi R (2009). ENETS Consensus Guidelines for the Standards of Care in
Neuroendocrine Tumors: peptide receptor radionuclide therapy with
radiolabeled somatostatin analogs. Neuroendocrinology.

[8] Ramage JK, Ahmed A, Ardill J (2012). Guidelines for the management of gastroenteropancreatic
neuroendocrine (including carcinoid) tumours (NETs). Gut.

[9] Koukouraki S, Strauss LG, Georgoulias V (2006). Evaluation of the pharmacokinetics of 68Ga-DOTATOC in patients
with metastatic neuroendocrine tumours scheduled for 90Y-DOTATOC
therapy. Eur J Nucl Med Mol Imaging.

[10] Buchmann I, Henze M, Engelbrecht S (2007). Comparison of 68Ga-DOTATOC and 111In-DTPAOC (Octreoscan) SPECT in
patients with neuroendocrine tumours. Eur J Nucl Med Mol Imaging.

[11] Gabriel M, Decristoforo C, Kendler D (2007). 68Ga-DOTA-Tyr3-octreotide PET in neuroendocrine tumors:
comparison with somatostatin receptor scintigraphy and CT. J Nucl Med.

[12] Krausz Y, Freedman N, Rubinstein R (2011). ^68^Ga-DOTA-NOC PET/ CT imaging of neuroendocrine
tumors: comparison with ^111^In-DTPA-octreotide
(OctreoScan^®^). Mol Imaging Biol.

[13] Naswa N, Sharma P, Kumar A (2011). Gallium-68-DOTA-NOC PET/ CT of patients with
gastroenteropancreatic neuroendocrine tumors: a prospective single-center
study. AJR Am J Roentgenol.

[14] Ambrosini V, Campana D, Tomassetti P (2011). PET-CT with 68Gallium-DOTA-peptides in NET: an
overview. Eur J Radiol.

[15] Geijer H, Breimer LH (2013). Somatostatin receptor PET/CT in neuroendocrine tumours: update on
systematic review and meta-analysis. Eur J Nucl Med Mol Imaging.

[16] Mojtahedi A, Thamake S, Tworowska I (2014). The value of (68)Ga-DOTATATE PET/CT in diagnosis and management
of neuroendocrine tumors compared to current FDA approved imaging
modalities: a review of literature. Am J Nucl Med Mol Imaging.

[17] Herrmann K, Czernin J, Wolin EM (2015). Impact of 68Ga-DOT-ATATE PET/CT on the management of
neuroendocrine tumors: the referring physician’s perspective. J Nucl Med.

[18] Skoura E, Michopoulou S, Mohmaduvesh M (2016). The impact of 68Ga-DOTATATE PET/CT imaging on management of
patients with neuroendocrine tumors: experience from a National Referral
Center in the United Kingdom. J Nucl Med.

[19] Sadowski SM, Neychev V, Millo C (2016). Prospective study of 68Ga-DOTATATE positron emission
tomography/computed tomography for detecting gastro-entero-pancreatic
neuroendocrine tumors and unknown primary sites. J Clin Oncol.

[20] Deppen SA, Blume J, Bobbey AJ (2016). ^68^Ga-DOTATATE compared with
^111^In-DTPA-Octreotide and conventional imaging for pulmonary and
gastroenteropancreatic neuroendocrine tumors: a systematic review and
meta-analysis. J Nucl Med.

[21] Barrio M, Czernin J, Fanti S (2017). The impact of somatostatin receptor-directed PET-CT on the
management of patients with neuroendocrine tumor: a systematic review and
meta-analysis. J Nucl Med.

[22] Subramaniam RM, Bradshaw ML, Lewis K (2018). ACR practice parameter for the performance of gallium-68 DOTATATE
PET/CT for neuroendocrine tumors. Clin Nucl Med.

[23] Ambrosini V, Campana D, Bodei L (2010). 68Ga-DOTANOC PET/ CT clinical impact in patients with
neuroendocrine tumors. J Nucl Med.

[24] Kowalski J, Henze M, Schuhmacher J (2003). Evaluation of positron emission tomography imaging using
[68Ga]-DOTA-D Phe1-Tyr3-Octreotide in comparison to [111In]-DTPAOC SPECT.
First results in patients with neuroendocrine tumors. Mol Imaging Biol.

[25] Srirajaskanthan R, Kayani I, Quigley AM (2010). The role of 68Ga-DOTATATE PET in patients with neuroendocrine
tumors and negative or equivocal findings on 111In-DTPA-octreotide
scintigraphy. J Nucl Med.

[26] Hofman MS, Kong G, Neels OC (2012). High management impact of Ga-68-DOTATATE (GaTate) PET/CT for
imaging neuroendocrine and other somatostatin expressing
tumours. J Med Imaging Radiat Oncol.

[27] Tierney JF, Kosche C, Schadde E (2019). ^68^Gallium-DOTATATE positron emission
tomography-computed tomography (PET CT) changes management in a majority of
patients with neuroendocrine tumors. Surgery.

[28] Crown A, Rocha FG, Raghu P (2020). Impact of initial imaging with gallium-68 dotatate PET/CT on
diagnosis and management of patients with neuroendocrine
tumors. J Surg Oncol.

[29] Bural GG, Muthukrishnan A, Oborski MJ (2012). Improved benefit of SPECT/CT compared to SPECT alone for the
accurate localization of endocrine and neuroendocrine tumors. Mol Imaging Radionucl Ther.

[30] Sanli Y, Garg I, Kandathil A (2018). Neuroendocrine tumor diagnosis and management:
^68^Ga-DOTATATE PET/CT. AJR Am J Roentgenol.

[31] Tirosh A, Kebebew E (2018). The utility of ^68^Ga-DOTATATE positronemission
tomography/computed tomography in the diagnosis, management, follow-up and
prognosis of neuroendocrine tumors. Future Oncol.

